# Supplementary material for: Risk Factors for Non-Communicable Diseases in Refugees, Asylum Seekers, and Subsidiary Protection Beneficiaries Resettled or Relocated in Portugal Between 2015 and 2020
Source: Int J Environ Res Public Health. 2024 Nov 13;21(11):1505. doi: 10.3390/ijerph21111505 (PMC11594213; doi:10.3390/ijerph21111505)
Supplement: Supplementary file 1 [file ijerph-21-01505-s001.zip › ijerph-3237447-supplementary-File S1.pdf]

Supplementary Material

Survey Information

Location and Date	Response	Code
Cluster/Centre/Village ID	|__|__|__|__|__|__|	I1
Cluster/Centre/Village name		I2
Interviewer ID	|__|__|__|	I3
Date of completion of the instrument	|__|__| |__|__| |__|__|__|__| dd mm year	I4

Consent, Interview Language and Name	Response	Code
Consent has been read and obtained	Yes 1<br>No 2 **If NO, END**	I5
Interview Language *[Insert Language]*	English 1<br>*[Add others]* 2<br>*[Add others]* 3<br>*[Add others]* 4	I6
Time of interview<br>(24-hour clock)	|__|__| : |__|__| hrs mins	I7
Family Surname		I8
First Name		I9
**Additional Information that may be helpful**		
Contact phone number where possible		I10

Step 1 Demographic Information

**CORE: Demographic Information**		
**Question**	**Response**	**Code**
Sex (*Record Male / Female as observed)*	Male 1<br>Female 2	C1
What is your date of birth?<br>*Don't Know 77 77 7777*	└─┴─┘ └─┴─┘ └─┴─┴─┴─┘ *If Known, Go to C4*<br>dd mm year	C2
How old are you?	Years └─┴─┘	C3
In total, how many years have you spent at school and in full-time study (excluding pre-school)?	Years └─┴─┘	C4

**EXPANDED: Demographic Information**		
What is the **highest level of education** you have completed?<br>*[INSERT COUNTRY-SPECIFIC CATEGORIES]*	No formal schooling 1<br>Less than primary school 2<br>Primary school completed 3<br>Secondary school completed 4<br>High school completed 5<br>College/University completed 6<br>Post graduate degree 7<br>Refused 88	C5
What is your *[insert relevant ethnic group / racial group / cultural subgroup / others]* **background**?	*[Locally defined]* 1<br>*[Locally defined]* 2<br>*[Locally defined]* 3<br>Refused 88	C6
What is your **marital status**?	Never married 1<br>Currently married 2<br>Separated 3<br>Divorced 4<br>Widowed 5<br>Cohabitating 6	C7

	Refused 88	
Which of the following best describes your **main work** status over the past 12 months?<br><br>*[INSERT COUNTRY-SPECIFIC CATEGORIES]*<br><br>(*USE SHOWCARD)*	Government employee 1<br>Non-government employee 2<br>Self-employed 3<br>Non-paid 4<br>Student 5<br>Homemaker 6<br>Retired 7<br>Unemployed (able to work) 8<br>Unemployed (unable to 9<br>Refused 88	C8
How many people older than 18 years, including yourself, live in your household?	Number of people └─┴─┘ *If Not Known, Go to C11*	C9

**EXPANDED: Demographic Information,** Continued			
**Question**	**Response**		**Code**
Taking **the past year**, can you tell me what the average earnings of the household have been?<br>*(RECORD ONLY ONE, NOT ALL 3)*	Per week └─┴─┴─┴─┴─┴─┴─┘	*Go to T1*	C10a
	OR per month └─┴─┴─┴─┴─┴─┴─┘	*Go to T1*	C10b
	OR per year └─┴─┴─┴─┴─┴─┴─┘	*Go to T1*	C10c
	Refused 88		C10d
Can you give an **estimate** of the annual household income if I read some options to you? Is it<br>*[INSERT QUINTILE VALUES IN LOCAL CURRENCY]*<br>*(READ OPTIONS)*	Quintile (Q) 1 1<br>More than Q 1, Q 2 2<br>More than Q 2, Q 3 3<br>More than Q 3, Q 4 4<br>More than Q 4 5<br>Don't Know 77<br>Refused 88		C11

Step 1 Behavioural Measurements

**CORE: Tobacco Use**			
Now I am going to ask you some questions about tobacco use.			
**Question**	**Response**		**Code**
Do you **currently** smoke any **tobacco** products, such as cigarettes, cigars or pipes?<br>*(USE SHOWCARD)*	Yes 1<br>No 2	*If No, go to T8*	T1
Do you currently smoke tobacco products **daily**?	Yes 1<br>No 2		T2
How old were you when you **first started** smoking?	Age (years)<br>Don't know 77 └─┴─┘	*If Known, go to T5a/T5aw*	T3
Do you remember how long ago it was?<br>*(RECORD ONLY 1, NOT ALL 3)*<br>*Don't know 77*	In Years └─┴─┘	*If known, go to T5a/T5aw*	T4a
	OR in Months └─┴─┘	*If known, go to T5a/T5aw*	T4b

	OR in Weeks └─┴─┘	T4c
On average, **how many** of the following products do you smoke **each day/week?**<br><br>*(IF LESS THAN DAILY, RECORD WEEKLY)*<br><br>*(RECORD FOR EACH TYPE, USE SHOWCARD)*<br><br>*Don't Know 7777*	DAILY↓ WEEKLY↓	
	Manufactured cigarettes └─┴─┴─┴─┘ └─┴─┴─┴─┘	T5a/T5aw
	Hand-rolled cigarettes └─┴─┴─┴─┘ └─┴─┴─┴─┘	T5b/T5bw
	Pipes full of tobacco └─┴─┴─┴─┘ └─┴─┴─┴─┘	T5c/T5cw
	Cigars, cheroots, cigarillos └─┴─┴─┴─┘ └─┴─┴─┴─┘	T5d/T5dw
	Number of Shisha sessions └─┴─┴─┴─┘ └─┴─┴─┴─┘	T5e/T5ew
	Other └─┴─┴─┴─┘ └─┴─┴─┴─┘<br>*If Other, go to T5other, else go to T6*	T5f/T5fw
	Other (please specify): └─┴─┴─┴─┴─┴─┘	T5other/<br>T5otherw
During the past 12 months, have you tried to **stop smoking**?	Yes 1<br>No 2	T6
During any visit to a doctor or other health worker in the past 12 months, were you advised to quit smoking tobacco?	Yes 1 *If T2=Yes, go to T12; if T2=No, go to T9*<br>No 2 *If T2=Yes, go to T12; if T2=No, go to T9*<br>No visit during the past 12 months 3 *If T2=Yes, go to T12; if T2=No, go to T9*	T7
In the past, did you **ever smoke** any tobacco products? *(USE SHOWCARD)*	Yes 1<br>No 2 *If no, go to T12*	T8
In the past, did you **ever** smoke **daily**?	Yes 1 If *T1=Yes, go to T12, else go to T10*<br>No 2 *If T1=Yes, go to T12, else go to T10*	T9

**EXPANDED: Tobacco Use**		
**Question**	**Response**	**Code**
How old were you when you **stopped** smoking?	Age (years)<br>Don't Know 77 └─┴─┘ *If known, go to T12*	T10
How **long ago** did you stop smoking?	Years ago, └─┴─┘ *If known, go to T12*	T11a
(RECORD ONLY 1, NOT ALL 3)	OR Months ago, └─┴─┘ *If known, go to T12*	T11b
Don't Know 77	OR Weeks ago, └─┴─┘	T11c
Do you **currently use** any **smokeless tobacco** products such as *[snuff, chewing tobacco, betel]*? *(USE SHOWCARD)*	Yes 1<br>No 2 *If no, go to T15*	T12
Do you **currently use smokeless tobacco** products **daily?**	Yes 1<br>No 2 *If no, go to T14aw*	T13
On average, how many **times a day/week** do you use ….<br>*(IF LESS THAN DAILY, RECORD WEEKLY)*<br>*(RECORD FOR EACH TYPE, USE SHOWCARD)*<br>*Don't Know 7777*	DAILY↓ WEEKLY↓	
	Snuff, by mouth └─┴─┴─┴─┘ └─┴─┴─┴─┘	T14a/<br>T14aw
	Snuff, by nose └─┴─┴─┴─┘ └─┴─┴─┴─┘	T14b/<br>T14bw
	Chewing tobacco └─┴─┴─┴─┘ └─┴─┴─┴─┘	T14c/<br>T14cw
	Betel, quid └─┴─┴─┴─┘ └─┴─┴─┴─┘	T14d/<br>T14dw
	Other └─┴─┴─┴─┘ └─┴─┴─┴─┘<br>*If Other, go to T14other, if T13=No, go to T16, else go to T17*	T14e/<br>T14ew
	Other (please specify): └─┴─┴─┴─┴─┴─┘<br>*If T13=No, go to T16, else go to T17*	T14other / T14otherw
In the **past**, did you **ever use** smokeless tobacco products such as *[snuff, chewing tobacco, or betel]*?	Yes 1<br>No 2 *If no, go to T17*	T15

In the **past**, did you **ever use** smokeless tobacco products such as *[snuff, chewing tobacco, or betel]* **daily**?	Yes 1<br>No 2	T16
During the past 30 days, did someone smoke **in your home**?	Yes 1<br>No 2	T17
During the past 30 days, did someone smoke in closed areas **in your workplace** (in the building, in a work area or a specific office)?	Yes 1<br>No 2<br>Don't work in a closed area 3	T18

**CORE: Alcohol Consumption**		
The next questions ask about the consumption of alcohol.		
**Question**	**Response**	**Code**
Have you **ever** consumed any alcohol such as beer, wine, spirits or *[add other local examples]*?<br>*(USE SHOWCARD OR SHOW EXAMPLES)*	Yes 1<br>No 2 *If no, go to A16*	A1
Have you consumed any alcohol within the **past 12 months**?	Yes 1 *If yes, go to A4*<br>No 2	A2
Have you stopped drinking due to health reasons, such as a negative impact on your health or on the advice of your doctor or other health worker?	Yes 1 *If Yes, go to A16*<br>No 2 *If no, go to A16*	A3
During the past 12 months, **how frequently** have you had at least one standard alcoholic drink?<br>*(READ RESPONSES, USE SHOWCARD)*	Daily 1<br>5-6 days per week 2<br>3-4 days per week 3<br>1-2 days per week 4<br>1-3 days per month 5<br>Less than once a month 6<br>Never 7	A4
Have you consumed any alcohol within the **past 30 days**?	Yes 1<br>No 2 *If no, go to A13*	A5
During the past 30 days, on how many **occasions** did you have at least one standard alcoholic drink?	Number<br>Don't know 77 └─┴─┘ *If zero, go to A13*	A6
During the past 30 days, when you drank alcohol, how many **standard drinks on average** did you have during one drinking occasion?<br>*(USE SHOWCARD)*	Number<br>Don't know 77 └─┴─┘	A7
During the past 30 days, what was the **largest number** of standard drinks you had on a single occasion, counting all types of alcoholic drinks together?	Largest number<br>Don't Know 77 └─┴─┘	A8
During the past 30 days, how many times did you have<br>**six or more** standard drinks in a single drinking occasion?	Number of times<br>Don't Know 77 └─┴─┘	A9
During each of the **past 7 days**, how many standard drinks did you have each day?	Monday └─┴─┘	A10a
	Tuesday └─┴─┘	A10b

(USE SHOWCARD)<br><br>*Don't Know 77*	Wednesday |__|__|	A10c
	Thursday |__|__|	A10d
	Friday |__|__|	A10e
	Saturday |__|__|	A10f
	Sunday |__|__|	A10g

**CORE: Alcohol Consumption, continued**		
I have just asked you about your consumption of alcohol during the past 7 days. The questions were about alcohol in general, while the next questions refer to your consumption of homebrewed alcohol, alcohol brought over the border/from another country, any alcohol not intended for drinking or other untaxed alcohol. Please only think about these types of alcohol when answering the next questions.		
**Question**	**Response**	**Code**
During the **past 7 days**, did you consume any **homebrewed** alcohol, any alcohol **brought over the border/from another country**, any alcohol **not intended for drinking** or other **untaxed** alcohol?<br><br>*[AMEND ACCORDING TO LOCAL CONTEXT]*<br><br>*(USE SHOWCARD)*	Yes 1<br><br>No 2 *If no, go to A13*	A11
On average, **how many standard drinks** of the following did you consume **during the past 7 days**?<br><br>*[INSERT COUNTRY-SPECIFIC EXAMPLES]*<br><br>*(USE SHOWCARD)*<br><br>*Don't Know 77*	Homebrewed spirits, e.g., moonshine |__|__|	A12a
	Homebrewed beer or wine, e.g., beer, palm or fruit wine |__|__|	A12b
	Alcohol brought over the border/from another country |__|__|	A12c
	Alcohol not intended for drinking, e.g., alcohol-based medicines, perfumes, after shaves |__|__|	A12d
	Other untaxed alcohol in the country |__|__|	A12e

**EXPANDED: Alcohol Consumption**			
During the **past 12 months,** how often have you found that you were not able to stop drinking once you had started?	Daily or almost daily<br>Weekly<br>Monthly<br>Less than monthly<br>Never	1<br>2<br>3<br>4<br>5	A13
During the **past 12 months,** how often have you failed to do what was normally expected from you because of drinking?	Daily or almost daily<br>Weekly<br>Monthly<br>Less than monthly<br>Never	1<br>2<br>3<br>4<br>5	A14
During the **past 12 months,** how often have you needed a first drink in the morning to get yourself going after a heavy drinking session?	Daily or almost daily<br>Weekly<br>Monthly<br>Less than monthly<br>Never	1<br>2<br>3<br>4<br>5	A15
During the **past 12 months**, have you had family problems or problems with your partner due to **someone else's** drinking?	Yes, more than monthly<br>Yes, monthly<br>Yes, several times but less than monthly<br>Yes, once or twice<br>No	1<br>2<br>3<br>4<br>5	A16

**CORE: Diet**		
The next questions ask about the fruits and vegetables that you usually eat. I have a nutrition card here that shows you some examples of local fruits and vegetables. Each picture represents the size of a serving. As you answer these questions please think of a typical week in the last year.		
**Question**	**Response**	**Code**
In a typical week, on how many days do you **eat fruit**? *(USE SHOWCARD)*	Number of days<br>Don't Know 77	
How many **servings** of fruit do you eat on **one** of those days? *(USE SHOWCARD)*	Number of servings<br>Don't Know 77 └─┴─┘	
In a typical week, on how many days do you **eat vegetables**? *(USE SHOWCARD)*	Number of days<br>Don't Know 77	D3
How many **servings** of vegetables do you eat on one of those days? *(USE SHOWCARD)*	Number of servings<br>Don't know 77 └─┴─┘	D4
5. Dietary salt		
6. With the next questions, we would like to learn more about salt in your diet. Dietary salt includes ordinary table salt, unrefined salt such as sea salt, iodized salt, salty stock cubes and powders, and salty sauces such as soy sauce or fish sauce (see showcard). The following questions are on adding salt to the food right before you eat it, on how food is prepared in your home, on eating processed foods that are high in salt such as [insert country specific examples], and questions on controlling your salt intake. Please answer the questions even if you consider yourself to eat a diet low in salt.		
How often do you **add salt or a salty sauce such as soy sauce** to your food right before you eat it or as you are eating it? *(SELECT ONLY ONE)*	Always 1<br>Often 2<br>Sometimes 3<br>Rarely 4<br>Never 5<br>Don't know 77	D5
How often is **salt, salty seasoning or a salty sauce added** in cooking or preparing foods in your household?	Always 1<br>Often 2<br>Sometimes 3<br>Rarely 4<br>Never 5<br>Don't know 77	D6

How often do you eat **processed food high in salt**? By processed food high in salt, I mean foods that have been altered from their natural state, such as packaged salty snacks, canned salty food including pickles and preserves, salty food prepared at a fast-food restaurant, cheese, bacon and processed meat *[add country specific examples].*	Always 1<br>Often 2<br>Sometimes 3<br>Rarely 4<br>Never 5<br>Don't know 77	D7
**How much salt or salty sauce** do you think you consume?	Far too much 1<br>Too much 2<br>Just the right amount 3<br>Too little 4<br>Far too little 5<br>Don't know 77	D8

**EXPANDED: Diet**		
**Question**	**Response**	**Code**
How important to you is **lowering the salt** in your	Very important 1<br>Somewhat important 2<br>Not at all important 3<br>Don't know 77	D9
Do you think that too much salt or salty sauce in your diet could cause a **health problem**?	Yes 1<br>No 2<br>Don't know 77	D10
Do you do any of the following on a regular basis to **control your salt intake**?<br>*(RECORD FOR EACH)*		
Limit consumption of processed foods	Yes 1<br>No 2	D11a
Look at the salt or sodium content on food labels	Yes 1<br>No 2	D11b
Buy low salt/sodium alternatives	Yes 1<br>No 2	D11c
Use spices other than salt when cooking	Yes 1<br>No 2	D11d
Avoid eating foods prepared outside of a home	Yes 1<br>No 2	D11e
Do other things specifically to control your salt intake	Yes 1 *If yes, go to*<br>No 2	D11f
Other (please specify)	|__|__|__|__|__|__|__|	D11other

**CORE: Physical Activity**		
Next I am going to ask you about the time you spend doing different types of physical activity in a typical week. Please answer these questions even if you do not consider yourself to be a physically active person.<br>Think first about the time you spend doing work. Think of work as the things that you have to do such as paid or unpaid work, study/training, household chores, harvesting food/crops, fishing or hunting for food, seeking employment. *[Insert other examples if needed].* In answering the following questions 'vigorous-intensity activities' are activities that require hard physical effort and cause large increases in breathing or heart rate, 'moderate-intensity activities' are activities that require moderate physical effort and cause small increases in breathing or heart rate.		
**Question**	**Response**	**Code**
**Work**		
Does your work involve vigorous-intensity activity that causes large increases in breathing or heart rate like *[carrying or lifting heavy loads, digging or construction work]* for at least 10 minutes continuously?	Yes 1<br>No 2 *If no, go to P 4*	
In a typical week, on how many days do you do vigorous-intensity activities as part of your work?	Number of days └─┘	
How much time do you spend doing vigorous-intensity activities at work on a typical day?	Hours: minutes └─┴─┘ : └─┴─┘ hrs mins	P3 (a-b)
Does your work involve moderate-intensity activity, that causes small increases in breathing or heart rate such as brisk walking *[or carrying light loads]* for at least 10 minutes continuously?	Yes 1<br>No 2 *If no, go to P 7*	P4
In a typical week, on how many days do you do moderate-intensity activities as part of your work?	Number of days └─┘	P5
How much time do you spend doing moderate-intensity activities at work on a typical day?	Hours: minutes └─┴─┘ : └─┴─┘ hrs mins	P6 (a-b)
**Travel to and from places**		
The next questions exclude the physical activities at work that you have already mentioned.<br>Now I would like to ask you about the usual way you travel to and from places. For example, to work, for shopping, to market, to place of worship. *[Insert other examples if needed]*		
Do you walk or use a bicycle *(pedal cycle)* for at least 10 minutes continuously to get to and from places?	Yes 1<br>No 2 *If no, go to P 10*	P7

In a typical week, on how many days do you walk or bicycle for at least 10 minutes continuously to get to and from places?	Number of days └─┘	P8
How much time do you spend walking or bicycling for travel on a typical day?	Hours: minutes └─┴─┘ : └─┴─┘ hrs mins	P9 (a-b)

**CORE: Physical Activity, Continued**		
**Question**	**Response**	**Code**
**Recreational activities**		
The next questions exclude the work and transport activities that you have already mentioned.<br><br>Now I would like to ask you about sports, fitness and recreational activities (leisure), *[Insert relevant terms]*.		
Do you do any vigorous-intensity sports, fitness or recreational *(leisure)* activities that cause large increases in breathing or heart rate like *[running or football]* for at least 10 minutes continuously?	Yes 1<br><br>No 2 *If no, go to P 13*	P10
In a typical week, on how many days do you do vigorous-intensity sports, fitness or recreational *(leisure)* activities?	Number of days └─┘	P11
How much time do you spend doing vigorous-intensity sports, fitness or recreational activities on a typical day?	Hours: minutes └─┴─┘ : └─┴─┘<br>hrs mins	P12<br>(a-b)
Do you do any moderate-intensity sports, fitness or recreational *(leisure)* activities that cause a small increase in breathing or heart rate such as brisk walking, *[cycling, swimming, volleyball]* for at least 10 minutes continuously?	Yes 1<br><br>No 2 *If no, go to P16*	P13
In a typical week, on how many days do you do moderate-intensity sports, fitness or recreational *(leisure)* activities?	Number of days └─┘	P14
How much time do you spend doing moderate-intensity sports, fitness or recreational *(leisure)* activities on a typical day?	Hours: minutes └─┴─┘ : └─┴─┘<br>hrs mins	P15<br>(a-b)

**EXPANDED: Physical Activity**		
**Sedentary behavior**		
The following question is about sitting or reclining at work, at home, getting to and from places, or with friends including time spent sitting at a desk, sitting with friends, traveling in car, bus, train, reading, playing cards or watching television, but do not include time spent sleeping.<br><br>*[INSERT EXAMPLES] (USE SHOWCARD)*		
How much time do you usually spend sitting or reclining on a typical day?	Hours: minutes └─┴─┘ : └─┴─┘<br>hrs mins	P16<br>(a-b)

**CORE: History of Raised Blood Pressure**		
**Question**	**Response**	**Code**
Have you ever had your blood pressure measured by a doctor or other health worker?	Yes 1<br>No 2 *If no, go to H6*	H1
Have you ever been told by a doctor or other health worker that you have raised blood pressure or hypertension?	Yes 1<br>No 2 *If no, go to H6*	H2a
Were you first told in the past 12 months?	Yes 1<br>No 2	H2b
In the past two weeks, have you taken any drugs (medication) for raised blood pressure prescribed by a doctor or other health worker?	Yes 1<br>No 2	H3
Have you ever seen a traditional healer for raised blood pressure or hypertension?	Yes 1<br>No 2	H4
Are you currently taking any herbal or traditional remedy for your raised blood pressure?	Yes 1<br>No 2	H5

**CORE: History of Diabetes**		
Have you ever had your blood sugar measured by a doctor or other health worker?	Yes 1<br>No 2 *If no, go to H12*	H6
Have you ever been told by a doctor or other health worker that you have raised blood sugar or diabetes?	Yes 1<br>No 2 *If no, go to H12*	H7a
Were you first told in the past 12 months?	Yes 1<br>No 2	H7b
In the past two weeks, have you taken any drugs (medication) for diabetes prescribed by a doctor or other health worker?	Yes 1<br>No 2	H8
Are you currently taking insulin for diabetes prescribed by a doctor or other health worker?	Yes 1<br>No 2	H9
Have you ever seen a traditional healer for diabetes or raised blood sugar?	Yes 1<br>No 2	H10
Are you currently taking any herbal or traditional remedy for your diabetes?	Yes 1<br>No 2	H11

**CORE: History of Raised Total Cholesterol**		
**Question**	**Response**	
Have you ever had your cholesterol (fat levels in your blood) measured by a doctor or other health worker?	Yes 1<br>No 2 *If no, go to H17*	H12
Have you ever been told by a doctor or other health worker that you have raised cholesterol?	Yes 1<br>No 2 *If no, go to H17*	H13a
Were you first told in the past 12 months?	Yes 1<br>No 2	H13b
In the past two weeks, have you taken any oral treatment (medication) for raised total cholesterol prescribed by a doctor or other health worker?	Yes 1<br>No 2	H14
Have you ever seen a traditional healer for raised cholesterol?	Yes 1<br>No 2	H15
Are you currently taking any herbal or traditional remedy for your raised cholesterol?	Yes 1<br>No 2	H16

**CORE: History of Cardiovascular Diseases**		
Have you ever had a heart attack or chest pain from heart disease (angina) or a stroke (cerebrovascular accident or incident)?	Yes 1<br>No 2	H17
Are you currently taking aspirin regularly to prevent or treat heart disease?	Yes 1<br>No 2	H18
Are you currently taking statins (Lovastatin/Simvastatin/Atorvastatin or any other statin) regularly to prevent or treat heart disease?	Yes 1<br>No 2	H19

**CORE: Lifestyle Advice**		
**Question**	**Response**	**Code**
During the past 12 months, have you visited a doctor or other health worker?	Yes 1<br>No 2 *If no and C1=1, go to M1*	H20
9. During any of your visits to a doctor or other health worker in the past 12 months, were you advised to do any of the following?<br>*(RECORD FOR EACH)*		
Quit using tobacco or don't start	Yes 1<br>No 2	H20a

Reduce salt in your diet	Yes 1<br>No 2	H20b
Eat at least five servings of fruit and/or vegetables each day	Yes 1<br>No 2	H20c
Reduce fat in your diet	Yes 1<br>No 2	H20d
Start or do more physical activity	Yes 1<br>No 2	H20e
Maintain a healthy body weight or lose weight	Yes 1<br>No 2	H20f
Reduce sugary beverages in your diet	Yes 1 *If C1=1 go to M1*<br>No 2 *If C1=1 go to M1*	H20g

CORE (for women only): Cervical Cancer Screening

The next question asks about cervical cancer prevention. Screening tests for cervical cancer prevention can be done in different ways, including Visual Inspection with Acetic Acid/vinegar (VIA), pap smear and Human Papillomavirus (HPV) test. VIA is an inspection of the surface of the uterine cervix after acetic acid (or vinegar) has been applied to it. For both pap smear and HPV test, a doctor or nurse uses a swab to wipe from inside your vagina, take a sample and send it to a laboratory. It is even possible that you were given the swab yourself and asked to swab the inside of your vagina. The laboratory checks for abnormal cell changes if a pap smear is done, and for the HP virus if an HPV test is done.

Have you ever had a screening test for cervical cancer, using any of these methods described above?	Yes 1<br>No 2<br>Don't know 77	CX1

Step 2 Physical Measurements

**CORE: Blood Pressure**		
**Question**	**Response**	**Code**
Interviewer ID	└─┴─┴─┘	M1
Device ID for blood pressure	└─┴─┘	M2
Cuff size used	Small 1<br>Medium 2<br>Large 3	M3
Reading 1	Systolic (mmHg) └─┴─┴─┘	M4a
	Diastolic (mmHg) └─┴─┴─┘	M4b
Reading 2	Systolic (mmHg) └─┴─┴─┘	M5a
	Diastolic (mmHg) └─┴─┴─┘	M5b
Reading 3	Systolic (mmHg) └─┴─┴─┘	M6a
	Diastolic (mmHg) └─┴─┴─┘	M6b
During the past two weeks, have you been treated for raised blood pressure with drugs (medication) prescribed by a doctor or other health worker?	Yes 1<br>No 2	M7
**CORE: Height and Weight**		
**For women:** Are you pregnant?	Yes 1 If *Yes, go to M 16*<br>No 2	M8
Interviewer ID	└─┴─┴─┘	M9
Device IDs for height and weight	Height └─┴─┘<br>Weight └─┴─┘	M10a<br>M10b
Height	in Centimetres (cm) └─┴─┴─┘.└─┘	M11
Weight<br>*If too large for scale 666.6*	in Kilograms (kg) └─┴─┴─┘.└─┘	M12
**CORE: Waist**		
Device ID for waist	└─┴─┘	M13
Waist circumference	in Centimetres (cm) └─┴─┴─┘.└─┘	M14

**EXPANDED: Hip Circumference and Heart Rate**		
Hip circumference	in Centimeters (cm) └─┴─┴─┘.└─┘	M15
Heart Rate		
Reading 1	Beats per minute └─┴─┴─┘	M16a
Reading 2	Beats per minute └─┴─┴─┘	M16b
Reading 3	Beats per minute └─┴─┴─┘	M16c

Step 3 Biochemical Measurements

**CORE: Blood Glucose**		
**Question**	**Response**	**Code**
During the past 12 hours have you had anything to eat or drink, other than water?	Yes 1<br>No 2	B1
Technician ID	└─┴─┴─┘	B2
Device ID	└─┴─┘	B3
Time of day blood specimen taken (24-hour clock)	Hours: minutes └─┴─┘ : └─┴─┘<br>hrs mins	B4
Fasting blood glucose<br>*[CHOOSE ACCORDINGLY: MMOL/L OR MG/DL]*	mmol/l └─┴─┘ └─┴─┘<br>mg/dl └─┴─┴─┘.└─┘	B5
Today, have you taken insulin or other drugs (medication) that have been prescribed by a doctor or other health worker for raised blood glucose?	Yes 1<br>No 2	B6
**CORE: Blood Lipids**		
Device ID	└─┴─┘	B7
Total cholesterol<br>*[CHOOSE ACCORDINGLY: MMOL/L OR MG/DL]*	mmol/l └─┴─┘ └─┴─┘<br>mg/dl └─┴─┴─┘.└─┘	B8
During the past two weeks, have you been treated for raised cholesterol with drugs (medication) prescribed by a doctor or other health worker?	Yes 1<br>No 2	B9
**CORE: Urinary sodium and creatinine**		
Had you been fasting prior to the urine collection?	Yes 1	B10

	No 2	
Technician ID	└─┴─┴─┘	B11
Device ID	└─┴─┘	B12
Time of day urine sample taken (24-hour clock)	Hours: minutes └─┴─┘ : └─┴─┘ hrs mins	B13
Urinary sodium	mmol/l └─┴─┴─┘ └─┘	B14
Urinary creatinine	mmol/l └─┴─┘ └─┴─┘	B15

**EXPANDED: Triglycerides and HDL Cholesterol**		
**Question**	**Response**	**Code**
Triglycerides<br>*[CHOOSE ACCORDINGLY: MMOL/L OR MG/DL]*	mmol/l └─┴─┘ └─┴─┘<br>mg/dl └─┴─┴─┘ . └─┘	B16
HDL Cholesterol<br>*[CHOOSE ACCORDINGLY: MMOL/L OR MG/DL]*	mmol/l └─┘ . └─┴─┘<br>mg/dl └─┴─┴─┘ . └─┘	B17

ANEXO III

Survey Information

Location and Date	Response
Cluster/Centre/Village ID	└─┴─┴─┴─┴─┴─┘
Cluster/Centre/Village name	
Interviewer ID	└─┴─┴─┘
Date of completion of the instrument	└─┴─┘ └─┴─┘ └─┴─┴─┴─┘<br>dd mm year

Consent, Interview Language and Name	Response
Consent has been read and obtained	Yes 1<br>No 2 **If NO, END**
Interview Language *[Insert Language]*	English 1<br>*[Add others]* 2<br>*[Add others]* 3<br>*[Add others]* 4
Time of interview<br>(24-hour clock)	└─┴─┘ : └─┴─┘<br>hrs mins
Family Surname	
First Name	

Step 1 Demographic Information

CORE: Demographic Information		
**Question**	**Response**	
Sex (*Record Male / Female as observed)*	Male 1<br>Female 2	**Q1**
What is your date of birth?	|__|__| |__|__| |__|__|__|__| *If known, go to question* ***Q3***<br>dd mm year	**Q2**
How old are you?<br>*Help participant estimate their age by interviewing them about their recollection of widely known major events.*	Years |__|__|	**Q3**
What is your country of origin? [a]	Afghanistan 1<br>Eritrea 2<br>Iraq 3<br>Syria 4<br>other 5<br>Other specify:	**Q4**
In which country did you apply for asylum? [a]	Italy 1<br>Greece 2<br>Turkey 3<br>Other 5<br>Other specify:	**Q5**
What was the period between the country of entry and the relocation / resettlement in Portugal[a]	|__|__| |__|__| |__|__|__|__| dd mm year	**Q6**

	Don't know 91<br>Refused 92	
During this period where you were staying[a]	Refugee Camp 1<br>Room rent 2<br>House/Apatment 3<br>Airport 4<br>Embarcation 5<br>Other 6<br>Other specify:<br>Don't know 91<br>Refused 92	**Q7**
In total, how many years have you spent at school and in full-time study (excluding pre-school)?<br>*Record total number of years of education (excluding pre-school and kindergarten).*	Years └─┴─┘<br>Don't know 91<br>Refused 92	**Q8**

[a] Questões adicionadas ao questionário STEPS original

**EXPANDED: Demographic Information**		
What is the **highest level of education** you have completed? [b]<br>*If a person attended a few months of the first year of secondary school but did not complete the year, record "primary school completed". If a person only attended a few years of primary school, record "less than primary school".*<br>*Circle appropriate response*	No formal schooling 1<br>Less than primary school 2<br>Primary school completed 3<br>Less than secondary school 4<br>Secondary school completed 5<br>High school completed 6	**Q9**

		College/University completed 7<br>Post graduate degree 8<br>Don't know 91<br>Refused 92	
What is your **marital status**?<br>*Circle the appropriate response*		Never married 1<br>Currently married 2<br>Separated 3<br>Divorced 4<br>Widowed 5<br>Cohabitating 6<br>Refused 92	**Q10**
Which of the following best describes your **main work** status<br>*The purpose of this question is to help answer other questions such as whether or not health status contributes to unemployment, or whether people indifferent kinds of occupations may be confronted with different risk fators.*<br>*Circle appropriate response*	on the 12 months prior to leaving the country of origin?	Government employee 1<br>Non-government 2<br>Self-employed 3<br>Non-paid 4<br>Student 5<br>Homemaker 6<br>Retired 7<br>Unemployed (able to 8<br>Unemployed (unable to 9<br>Refused 92	**Q11**
	at present?	Government employee 1<br>Non-government 2<br>Self-employed 3<br>Non-paid 4<br>Student 5<br>Homemaker 6<br>Retired 7<br>Unemployed (able to 8<br>work)<br>9	**Q12**

[b] no formal school – 0 ano de escolaridade formal; less than primary school – menos de 4 anos de escolaridade; primary school competed – 4 anos de escolaridade completos; secondary school incompleted – entre 5 e 9 anos de escolaridade; secondary school completed – 9 anos de escolaridade; high school completed – 12 anos de escolaridade completos.

Can you tell me what is the average earnings?<br>*(RECORD ONLY ONE, NOT ALL 3)* [c]<br><br>*Record the average earnings by week, month, or year. If refused to answer, skip next question.*	on the 12 months prior to leaving the country of origin?	Per week ⎣_⎦_⎦_⎦_⎦_⎦_⎦_⎦ *Go to question* ***Q20***<br>OR per month ⎣_⎦_⎦_⎦_⎦_⎦_⎦_⎦ *Go to question* ***Q20***<br>OR per year ⎣_⎦_⎦_⎦_⎦_⎦_⎦_⎦ *Go to question* ***Q20***<br>Don't know 91<br>Refused 92	**Q13**
	at present?	Per week ⎣_⎦_⎦_⎦_⎦_⎦_⎦_⎦ *Go to question* ***Q20***<br>OR per month ⎣_⎦_⎦_⎦_⎦_⎦_⎦_⎦ *Go to question* ***Q20***<br>OR per year ⎣_⎦_⎦_⎦_⎦_⎦_⎦_⎦ *Go to question* ***Q20***<br>Don't know 91<br>Refused 92	**Q14**

[c] As questões do STEPS original "How many people older than 18 years, including yourself, live in your household?; "Can you tell me what the average earnings of the household have been?" and "Can you give an estimate of the annual household income if I read some options to you?"não foram realizadas e em sua substituição foi formulada a seguinte questão "Can you tell me what is your average earnings?".

Step 1 Behavioural Measurements

CORE: Tobacco Use		
Now I am going to ask you some questions about tobacco use at the present and on the 12 months prior to leaving the country of origin		
**Question**	**Response**	
Do you currently smoke any **tobacco** products, such as cigarettes, cigars, or pipes?<br>*(USE SHOWCARD)*<br>*Ask the participant to think of any tobacco products he/she is smoking since is recolocation or resettlement*	Yes 1<br>No 2 If *no, go to question* **Q25**<br>Don't know 91<br>Refused 92	**Q15**
Do you currently smoke tobacco products **daily?**<br>*This question is only for current smokers of tobacco products.*<br>*Ask the participant if he/she smoke tobbaco products daily, since is recolocation or resettlement*	Yes 1<br>No 2 If *No, go to question* **Q25**<br>Don't know 91<br>Refused 92	**Q16**
How old were you when you **first started** smoking daily?<br>*For current daily smokers only. Ask the participant to think of the time when he/she started to smoke any tobacco products daily.*	Age (years)<br>└─┴─┘ *If known, go to question* **Q19**<br>Don't know 91<br>Refused 92	**Q17**
Do you remember how long ago it	In Years └─┴─┘ *If Known, go to question*<br>OR in Months └─┴─┘ *If known, go to question*	**Q18**

<table>
<tr><td colspan="2">was?

(RECORD ONLY 1, NOT ALL 3)

If the participant doesn’t remember his/her age when started smoking, then record the time in years, months or weeks as appropriate.</td><td>OR in Weeks |__|__|
Don’t know 91

Refused 92</td><td></td></tr>
<tr><td rowspan="12">On average, how many of the following products do you smoke each day/week

(IF LESS THAN DAILY, RECORD WEEKLY)

(RECORD FOR EACH TYPE, USE SHOWCARD)

For current daily smokers only.

Specify zero if no products were used in</td><td rowspan="8">in the 12 months prior to leaving the country of origin?</td><td>DAILY↓ WEEKLY↓</td><td></td></tr>
<tr><td>Manufactured cigarettes |__|__|__|__| |__|__|__|__|</td><td>Q19a</td></tr>
<tr><td>Hand-rolled cigarettes |__|__|__|__| |__|__|__|__|</td><td>Q19b</td></tr>
<tr><td>Pipes full of tobacco |__|__|__|__| |__|__|__|__|</td><td>Q19c</td></tr>
<tr><td>Cigars, cheroots, cigarillos |__|__|__|__| |__|__|__|__|</td><td>Q19d</td></tr>
<tr><td>Other |__|__|__|__| |__|__|__|__|
If Other, go to question Q19other</td><td>Q19e</td></tr>
<tr><td>Other (please specify): |__|__|__|__|__|__|</td><td>Q19 other</td></tr>
<tr><td>Don’t know 91</td><td>Q19f</td></tr>
<tr><td rowspan="5">at present?</td><td>Refused 92</td><td>Q19g</td></tr>
<tr><td>Manufactured cigarettes |__|__|__|__| |__|__|__|__|</td><td>Q20a</td></tr>
<tr><td>Hand-rolled cigarettes |__|__|__|__| |__|__|__|__|</td><td>Q20b</td></tr>
<tr><td>Pipes full of tobacco |__|__|__|__| |__|__|__|__|</td><td>Q20c</td></tr>
<tr><td>Cigars, cheroots, cigarillos |__|__|__|__| |__|__|__|__|</td><td>Q20d</td></tr>
</table>

each category<br>*instead of leaving categories blank. Then go to question.*<br>*Daily smokers don't have to answer questions on past*<br>*smoking question - question.*		Other ⌊_⌋_⌋_⌋_⌋ ⌊_⌋_⌋_⌋_⌋<br>*If Other, go to question **Q20 other***	**Q20e**
		Other (please specify): ⌊_⌋_⌋_⌋_⌋_⌋_⌋	**Q20**<br>**other**
		Don't know 91	**Q20f**
		Refused 92	**Q20g**
Have you tried to **stop smoking**	in the 12 months prior to leaving the country of origin?	Yes 1<br>No 2<br>Don't know 91<br>Refused 92	**Q21**
	at present?	Yes 1<br>No 2<br>Don't know 91<br>Refused 92	**Q22**
During any visit to a doctor or other health worker where you advised to quit smoking tobacco?	in the 12 months prior to leaving the country of origin?	Yes 1 *If **Q16**=Yes, go to question **Q29**; if **Q16**=No, go to question **Q25***<br>No 2 *If **Q16**=Yes, go to question **Q29**; if **Q16**=No, go to question **Q25***<br>No visit during the past 12 months 3 *If **Q16**=Yes, go to question **Q29**; if **Q16**=No, go to question **Q25***<br>Don't know 91<br>Refused 92	**Q23**
	at present?	Yes 1 *If **Q17**=Yes, go to question **Q30**; if **Q17**=No, go to question **Q26***<br>No 2 *If **Q17**=Yes, go to question **Q30**; if **Q17**=No, go to question **Q26***<br>No visit during the past 12 months 3 *If **Q17**=Yes, go to question **Q30**; if **Q17**=No, go to question **Q26***<br>Don't know 91<br>Refused 92	**Q24**
In the past did you **ever smoke** any tobacco products?<br>*(USE SHOWCARD)*		Yes 1<br>No 2 If *no, go to **Q33***<br>Don't know 91	**Q25**

in the 12 months prior to leaving the country of origin	Refused 92	
In the past did you **ever** smoke **daily**?<br><br>*in the 12 months prior to leaving the country of origin*	Yes 1 If ***Q15****=Yes, go to* ***Q34/Q35****, else go to* ***Q27***<br>No 2 *If* ***Q15****=Yes, go to* ***Q34/Q35****, else go to* ***Q27***<br>Don't know 91<br>Refused 92	**Q26**

**EXPANDED: Tobacco Use**		
**Question**	**Response**	
How old were you when you **stopped** smoking?<br><br>*Ask the participant to think of the time when he/she stopped smoking tobacco products on a daily basis.*	Age (years) └─┴─┘ *If known, go to* ***Q29 and Q30***<br>Don't Know 91 *If known, go to* ***Q29 and Q30***<br>Refused 92 *If refused, go to* ***Q29 and Q30***	**Q27**
How **long ago** did you stop smoking?<br><br>*(RECORD ONLY 1, NOT ALL 3)*<br><br>*If the participant doesn't remember his/her age when*<br>*they started smoking, then record the time in weeks,*<br>*months or years as appropriate.*	Years ago, └─┴─┘ *If known, go to* ***Q29 and Q30***<br>Don't Know 91 *If known, go to* ***Q29 and Q30***<br>Refused 92 *If refused, go to* ***Q29 and Q30***<br>OR Months ago, └─┴─┘ *If Known, go to* ***Q29 and Q30***	**Q28**

		Don't Know 91	*If Known, go to **Q29 and Q30***	
		Refused 92	*If refused, go to **Q29 and Q30***	
		OR Weeks ago,	└─┴─┘ *If known, go to **Q29 and Q30***	
		Don't Know	91 *If known, go to **Q29 and Q30***	
		Refused	92 *If refused, go to **Q29 and Q30***	
Do you **use any smokeless tobacco** products such as *[snuff, chewing tobacco, betel] (USE SHOWCARD)*	in the 12 months prior to leaving the country of origin)	Yes	1	**Q29**
		No	2 If NO, go to **Q33**	
		Don't Know	91	
		Refused	92	
	at present?	Yes	1	**Q30**
		No	2 If NO, go to **Q33**	
		Don't Know	91	
		Refused	92	
Do you **use smokeless tobacco** products **daily**	in the 12 months prior to leaving the country of origin)	Yes	1	**Q31**
		No	2	
		Don't Know	91	
		Refused	92	
	at present?	Yes	1	**Q32**
		No	2	

		Don't Know 91<br>Refused 92	
Did someone smoke **in your home**	in the 12 months prior to leaving the country of origin?	Yes 1<br>No 2<br>Don't Know 91<br>Refused 92	**Q33**
	at present?	Yes 1<br>No 2<br>Don't Know 91<br>Refused or don´t [illegible] 92	**Q34**
Did someone smoke in closed areas **in your workplace** (in the building, in a work area or a specific office)	In the 12 months prior to leaving the country of origin?	Yes 1<br>No 2<br>Don't Know 91<br>Refused or don't Wor in a closed area 92	**Q35**
	at present?	Yes 1<br>No 2<br>Don't Know 91<br>Refused 92	**Q36**

**CORE: Alcohol Consumption**			
The next questions ask about the consumption of alcohol at the present and on the 12 months prior to leaving the country of origin			
**Question**		**Response**	
Have you **ever** consumed any alcohol such as beer, wine, spirits?<br><br>*(USE SHOWCARD OR SHOW EXAMPLES)*<br><br>*Think of any drinks that contain alcohol.*		Yes 1<br>No 2 ***If No, END Alcohol consumption***<br>Don't Know 91 ***If No, END Alcohol consumption***<br>Refused 92 ***If No, END Alcohol***	**Q37**
Have you consumed any alcohol?<br>*Think of any drinks that contain alcohol*	on the12 months prior to leaving the country of origin?	Yes 1 *If yes, go to* ***Q41***<br>No 2<br>Don't Know 91<br>Refused 92	**Q38**
	at present	Yes 1 *If yes, go to* ***Q41***<br>No 2<br>Don't Know 91<br>Refused 92	**Q39**
Have you stopped drinking due to health reasons, such as a negative impact on your health or on the advice of your doctor or other health worker?		Yes 1 ***If YES, END Alcohol consumption***<br>No 2<br>Don't Know 91<br>Refused 92	**Q40**
**How frequently** have	on the12 months	Daily 1	**Q41a**

you had at least one standard alcoholic drink?<br>*(READ RESPONSES, USE SHOWCARD)*	prior to leaving the country of origin?	5-6 days per week 2<br>3-4 days per week 3<br>1-2 days per week 4<br>1-3 days per month 5<br>Less than once a 6<br>Never 7<br>Don't Know 91<br>Refused 92	**Q41b**<br>**Q41c**<br>**Q41d**<br>**Q41e**<br>**Q41f**<br>**Q42g**<br>**Q42h**<br>**Q42i**
	at present?	Daily 1<br>5-6 days per week 2<br>3-4 days per week 3<br>1-2 days per week 4<br>1-3 days per month 5<br>Less than once a month 6<br>Never 7<br>Don't Know 91<br>Refused 92	**Q42a**<br>**Q42b**<br>**Q42c**<br>**Q42d**<br>**Q42e**<br>**Q42f**<br>**Q42g**<br>**Q42h**<br>**Q42i**
How many **occasions** did you have at least one standard alcoholic drink	on the 12 months prior to leaving the country of origin?	Number └─┴─┘ *If Zero, END*<br>Don't Know 91<br>Refused 92	**Q43**
	during the past 30 days?	Number └─┴─┘ *If Zero, END*<br>Don't Know 91<br>Refused 92	**Q44**
When you drank alcohol, how many **standard drinks on average** did you	on the 12 months prior to leaving the country of origin?	Number └─┴─┘<br>Don't Know 91	**Q45**

have during one drinking occasion<br>*(USE SHOWCARD)*		Refused 92	
	during the past 30 days	Number └┴┘<br>Don't Know 91<br>Refused 92	**Q46**
What was the **largest number** of standard drinks you had on a single occasion, counting all types of alcoholic drinks together	on the 12 months prior to leaving the country of origin?	Largest number └┴┘<br>Don't Know 91<br>Refused 92	**Q47**
	during the past 30 days?	Number of times └┴┘<br>Don't Know 91<br>Refused 92	**Q48**
How many times did you have **six or more** standard drinks in a single drinking occasion	on the 12 months prior to leaving the country of origin?	Number of times └┴┘<br>Don't Know 91<br>Refused 92	**Q49**
	during the past 30 days?	Number of times └┴┘<br>Don't Know 91<br>Refused 92	**Q50**
	in a typical week on the12 months prior to leaving the country of origin?	Monday └┴┘	**Q51a**
		Tuesday └┴┘	**Q51b**
		Wednesday └┴┘	**Q51c**
		Thursday └┴┘	**Q51d**

How many standard drinks did you have each day?<br><br>*(USE SHOWCARD)*		Friday	**Q51e**
		Saturday	**Q51f**
		Sunday	**Q51g**
		Don't Know 91	**Q51h**
		Refused 92	**Q51i**
	during the past 7 days?	Monday	**Q52a**
		Tuesday	**Q52b**
		Wednesday	**Q52c**
		Thursday	**Q52d**
		Friday	**Q52e**
		Saturday	**Q52f**
		Sunday	**Q52g**
		Don't Know 91	**Q52gh**
		Refused 92	**Q52i**

<table>
<tr><td colspan="4">CORE: Alcohol Consumption, continued</td></tr>
<tr><td colspan="4">I have just asked you about your consumption of alcohol during the past 7 days. The questions were about alcohol in general, while the next questions refer to your consumption of homebrewed alcohol, alcohol brought over the border/from another country, any alcohol not intended for drinking or other untaxed alcohol. Please only think about these types of alcohol when answering the next questions.</td></tr>
<tr><td colspan="2">Question</td><td>Response</td><td></td></tr>
<tr><td rowspan="2">Did you consume any homebrewed alcohol, any alcohol brought over the border/from another country, any alcohol not intended for drinking or other untaxed alcohol?
(USE SHOWCARD)</td><td>on the12 months prior to leaving the country of origin?</td><td>Yes 1
No 2 If NO, end
Don’t Know 91
Refused 92</td><td>Q53</td></tr>
<tr><td>during the past 7 days?</td><td>Yes 1
No 2 If NO, end
Don’t Know 91
Refused 92</td><td>Q54</td></tr>
<tr><td rowspan="4">On average, how many standard drinks of the following did you consume
(USE SHOWCARD)
Don’t Know 3</td><td rowspan="4">In a typical week on the12 months prior to leaving the country of origin?</td><td>Homebrewed spirits, e.g., moonshine |__|__|</td><td rowspan="4">Q55</td></tr>
<tr><td>Homebrewed beer or wine, e.g., beer, palm or fruit wine |__|__|</td></tr>
<tr><td>Alcohol brought over the border/from another country |__|__|</td></tr>
<tr><td>Alcohol not intended for drinking, e.g., alcohol-based medicines, perfumes, |__|__|</td></tr>
</table>

		after shaves	
		Other untaxed alcohol in the country └─┴─┘<br>Don't Know 91<br>Refused 92	
	during the past 7 days?	Homebrewed spirits, e.g., moonshine └─┴─┘	**Q56**
		Homebrewed beer or wine, e.g. beer, palm or fruit wine └─┴─┘	
		Alcohol brought over the border/from another country └─┴─┘	
		Alcohol not intended for drinking, e.g., alcohol-based medicines, perfumes, after shaves └─┴─┘	
		Other untaxed alcohol in the country └─┴─┘<br>Don't Know 91<br>Refused 92	

**CORE: Diet**			
The next questions ask about the fruits and vegetables that you usually eat. I have a nutrition card here that shows you some examples of local fruits and vegetables. Each picture represents the size of a serving. As you answer these questions please think of a typical week in the present and on the 12 months prior to leaving the country of origin			
**Question**		**Response**	
In a typical week how, many days do you **eat fruit**<br>*(USE SHOWCARD)*<br><br>*Think of any fruit on the show card. A typical week means a "normal" week when your diet is not affected by cultural, religious, or other events. Do not report an average over a period.*	on the 12 months prior to leaving the country of origin?	Number of servings └─┴─┘ If Zero days, go to **Q61**<br>Don't Know 91<br>Refused 92	**Q57**
	at present?	Number of servings └─┴─┘ If Zero days, go to **Q62**<br>Don't Know 91<br>Refused 92	**Q58**
How many **servings** of fruit do you eat on **one** of those days *(USE SHOWCARD)*<br><br>*Think of one day the participant can recall easily*	on the 12 months prior to leaving the country of origin?	Number of servings └─┴─┘<br>Don't Know 91<br>Refused 92	**Q59**
	at present?	Number of servings └─┴─┘<br>Don't Know 91<br>Refused 92	**Q60**
In a typical week how, many days do you **eat vegetables**<br>*(USE SHOWCARD)*<br><br>*Think of any vegetable on the show card. A typical week means a "normal" week when your diet is not affected by cultural, religious, or other events. Do not report an average over a period.*	on the 12 months prior to leaving the country of origin?	Number of servings └─┴─┘ If Zero days, go to **Q65**<br>Don't Know 91<br>Refused 92	**Q61**
	at present?	Number of servings └─┴─┘ If Zero days, go to **Q65**<br>Don't Know 91	**Q62**

		Refused 92	
How many **servings** of vegetables do you eat on one of those days?<br><br>*(USE SHOWCARD)*<br><br>*Think of one day the participant can recall easily*	on the 12 months prior to leaving the country of origin?	Number of servings └─┴─┘<br><br>Don't Know 91<br><br>Refused 92	**Q63**
	at present?	Number of servings └─┴─┘<br><br>Don't Know 91<br><br>Refused 92	**Q64**

**CORE: Dietary salt**			
With the next questions, we would like to learn more about salt in your diet. Dietary salt includes ordinary table salt, unrefined salt such as sea salt, iodized salt, salty stock cubes and powders, and salty sauces such as soy sauce or fish sauce (see showcard). The following questions are on adding salt to the food right before you eat it, on how food is prepared in your home, on eating processed foods that are high in salt such as [insert country specific examples], and questions on controlling your salt intake. Please answer the questions even if you consider yourself to eat a diet low in salt.			
How often do you **add salt or a salty sauce such as soy sauce** to your food right before you eat it or as you are eating<br><br>*(SELECT ONLY ONE)*<br><br>*(USE SHOWCARD)*	on the 12 months prior to leaving the country of origin?	Always 1<br>Often 2<br>Sometimes 3<br>Rarely 4<br>Never 5<br>Don't Know 91<br><br>Refused 92	**Q65**
	at present?	Always 1<br>Often 2<br>Sometimes 3<br>Rarely 4<br>Never 5<br>Don't Know 91<br><br>Refused 92	**Q66**
How often is **salt, salty seasoning or a salty sauce added** in	on the 12 months prior to leaving the	Always 1<br>Often 2<br>Sometimes 3<br>Rarely 4	**Q67**

cooking or preparing foods in your household	country of origin?	Never 5<br>Don't Know 91<br>Refused 92	
	at present?	Always 1<br>Often 2<br>Sometimes 3<br>Rarely 4<br>Never 5<br>Don't Know 91<br>Refused 92	**Q68**
How often do you eat **processed food high in salt?**<br>By processed food high in salt, I mean foods that have been altered from their natural state, such as packaged salty snacks, canned salty food including pickles and preserves, salty food prepared at a fast food restaurant, cheese, bacon and processed meat<br>*(USE SHOWCARD)*	on the 12 months prior to leaving the country of origin?	Always 1<br>Often 2<br>Sometimes 3<br>Rarely 4<br>Never 5<br>Don't Know 91<br>Refused 92	**Q69**
	at present?	Always 1<br>Often 2<br>Sometimes 3<br>Rarely 4<br>Never 5<br>Don't Know 91<br>Refused 92	**Q70**
**How much salt or salty sauce** do you think you consume?	on the 12 months prior to leaving the country of origin?	Far too much 1<br>Too much 2<br>Just the right amount 3<br>Too little 4<br>Far too little 5<br>Don't Know 91<br>Refused 92	**Q71**
	at present?	Far too much 1<br>Too much 2<br>Just the right amount 3<br>Too little 4<br>Far too little 5<br>Don't Know 91<br>Refused 92	**Q72**

**CORE: Physical Activity**			
Next, I am going to ask you about the time you spend doing different types of physical activity in a typical week in the 12 months prior to leaving the country of origin and at present. Please answer these questions even if you do not consider yourself to be a physically active person. Think first about the time you spend doing work. Think of work as the things that you must do such as paid or unpaid work, study/training, household chores, harvesting food/crops, fishing or hunting for food, seeking employment. *[Insert other examples if needed].* In answering the following questions 'vigorous-intensity activities' are activities that require hard physical effort and cause large increases in breathing or heart rate, 'moderate-intensity activities' are activities that require moderate physical effort and cause small increases in breathing or heart rate.			
**Question**		**Response**	
**Work**			
Does your work involve vigorous-intensity activity that causes large increases in breathing or heart rate like *[carrying or lifting heavy loads, digging or construction work]* for at least 10 minutes continuously?<br>*(USE SHOWCARD)*	in the 12 months prior to leaving the country of origin?	Yes 1<br>No 2 *If no, go to question* ***Q79***<br>Don't Know 91 *If Don´t Know, go to question* ***Q79***<br>Refused 92	**Q73**
	at present?	Yes 1<br>No 2 *If no, go to question* ***Q80***<br>Don't Know 91 *If Don´t Know, go to question* ***Q80***<br>Refused 92	**Q74**
In a typical week, on how many days do you do vigorous-intensity activities as part of your work?<br>*Typical week" means a week when a person is doing vigorous intensity activities and not an average over a period. Valid responses range from 1-7.*	in the 12 months prior to leaving the country of origin?	Number of days └─┘<br>Don't know 91<br>Refused 92	**Q75**
	at present?	Number of days └─┘<br>Don't know 91<br>Refused 92	**Q76**
How much time do you spend doing	in the 12 months prior to leaving the	Hours: minutes └─┴─┘ : └─┴─┘	**Q77**

vigorous-intensity activities at work?<br><br>*Think of one day you can recall easily. Consider only those activities undertaken continuously for 10 minutes or more. Probe very high responses (over 4 hrs) to verify.*	country of origin?	hrs mins<br>Don't know 91<br>Refused 92	
	at present?	Hours: minutes └─┴─┘ : └─┴─┘<br>hrs mins<br>Don't know 91<br>Refused 92	**Q78**

Does your work involve moderate-intensity activity, that causes small increases in breathing or heart rate such as brisk walking *[or carrying light loads]* for at least 10 minutes continuously? *(USE SHOWCARD)*	in the 12 months prior to leaving the country of origin?	Yes 1<br>No 2 *If no, go to question **Q85***<br>Don't Know 91<br>Refused 92	**Q79**
	at present?	Yes 1<br>No 2 *If no, go to question **Q86***<br>Don't Know 91<br>Refused 92	**Q80**
In a typical week, on how many days do you do moderate-intensity activities as part of your work? *Valid responses range from 1-7*	in the 12 months prior to leaving the country of origin?	Number of days ⌞_⌟<br>Don't Know 91<br>Refused 92	**Q81**
	at present?	Number of days ⌞_⌟<br>Don't Know 91<br>Refused 92	**Q82**
How much time do you spend doing moderate-intensity activities at work on a typical day? *Think of one day you can recall easily. Consider only those activities undertaken*	in the 12 months prior to leaving the country of origin?	Hours: minutes ⌞_⌞_⌟ : ⌞_⌞_⌟ hrs mins<br>Don't know 91 Don't know 91<br>Refused 92 Refused 92	**Q83**

continuously for 10 minutes or more. Probe very high responses (over 4 hrs) to verify.	at present?	Hours: minutes └─┴─┘ : └─┴─┘ hrs mins<br>Don't know 91 Don't know 91<br>Refused 92 Refused 92	**Q84**
**Travel to and from places**			
The next questions exclude the physical activities at work that you have already mentioned. Now I would like to ask you about the usual way you travel to and from places. For example, to work, for shopping, to market, to place of worship in the 12 months prior to leaving the country of origin and at present.<br>*The introductory statement to the following questions on transport-related physical activity is very important. It asks and helps the participant to now think about how they travel around getting from place-to-place. This statement **should not** be omitted*			
Did you walk or use a bicycle *(pedal cycle)* for at least 10 minutes continuously to get to and from places	in the 12 months prior to leaving the country of origin?	Yes 1<br>No 2 *If no, go to question **Q91***<br>Don't Know 91<br>Refused 92	**Q85**
	at present?	Yes 1<br>No 2 *If no, go to question **Q92***<br>Don't Know 91<br>Refused 92	**Q86**
In a typical week, on how many days do you walk or bicycle for at least 10 minutes continuously to get to and from places<br>*Valid responses range from 1-7*	in the 12 months prior to leaving the country of origin?	Number of days └─┘<br>Don't Know 91<br>Refused 92	**Q87**
	at present?	Number of days └─┘<br>Don't Know 91<br>Refused 92	**Q88**

How much time do you spend walking or bicycling for travel on a typical day	in the 12 months prior to leaving the country of origin?	Hours: minutes └─┴─┘ : └─┴─┘ hrs mins<br>Don't Know 91<br>Refused 92	**Q89**
	at present?	Hours: minutes └─┴─┘ : └─┴─┘ hrs mins<br>Don't Know 91<br>Refused 92	**Q90**

CORE: Physical Activity, Continued

Recreational activities

The next questions exclude the work and transport activities that you have already mentioned. Now I would like to ask you about sports, fitness and recreational activities (leisure).

This introductory statement directs the participant to think about recreational activities. This can also be called discretionary or leisure time. It includes sports and exercise but is not limited to participation competitions. Activities reported should be done regularly and not just occasionally. It is important to focus on only recreational activities and not to include any activities already mentioned. This statement ***should not*** *be omitted.*

Do you do any vigorous-intensity sports, fitness or recreational *(leisure)* activities that cause large increases in breathing or heart rate like *[running or football]* for at least 10 minutes continuously?<br><br>*(USE SHOWCARD)*	in the 12 months prior to leaving the country of origin?	Yes 1<br>No 2 *If No, go to question **Q97***<br>Don't Know 91 *If Don't Know, go to question **Q97***<br>Refused 92 *If refused, go to question **Q97***	**Q91**
	at present?	Yes 1<br>No 2 *If No, go to question **Q98***<br>Don't Know 91 *If Don't Know, go to question **Q98***<br>Refused 92 *If Refused, go to question **Q98***	**Q92**
In a typical week how many days do	in the 12 months prior to leaving the	Number of days └─┘	**Q93**

you do vigorous-intensity sports, fitness or recreational *(leisure)* activities?<br><br>*Valid responses range from 1-7.*	country of origin?	Don't Know 91<br>Refused 92	
	at present?	Number of days __<br>Don't Know 91<br>Refused 92	**Q94**
How much time do you spend doing vigorous-intensity sports, fitness or recreational activities on a typical day?<br><br>*Think of one day you can recall easily. Consider the total amount of time doing vigorous recreational activities for periods of 10 minutes or more. Probe very high responses (over 4 hrs).*	in the 12 months prior to leaving the country of origin?	Hours: minutes __ : __ (hrs, mins)<br>Don't Know 91<br>Refused 92	**Q95**
	at present?	Hours: minutes __ : __ (hrs, mins)<br>Don't Know 91<br>Refused 92	**Q96**
Do you do any moderate-intensity sports, fitness or recreational *(leisure)* activities that cause a small increase in breathing or heart rate such as brisk walking, *[cycling, swimming, volleyball]*	in the 12 months prior to leaving the country of origin?	Yes 1<br>No 2 *If no, go to question* ***Q103***<br>Don't Know 91 *If Don´t Know, go to question* ***Q103***<br>Refused 92 *If Refused, go to question*	**Q97**
	at present?	Yes 1<br>No 2 *If no, go to question* ***Q104***<br>Don't Know 91 *If Don´t Know, go to question* ***Q104***<br>Refused 92 *If Refused, go to question*	**Q98**
In a typical week	in the 12 months	Number of days	**Q99**

how, many days do you do moderate-intensity sports, fitness or recreational *(leisure)* activities<br><br>*Valid responses range from 1-7*	prior to leaving the country of origin?	└─┘<br>Don't Know 91<br>Refused 92	
	at present?	Number of days └─┘<br>Don't Know 91<br>Refused 92	**Q100**
How much time do you spend doing moderate-intensity sports, fitness or recreational *(leisure)* activities on a typical day<br><br>*Think of one day you can recall easily. Consider the total amount of time doing moderate recreational activities for periods of 10 minutes or more. Probe very high responses (over 4 hrs).*	in the 12 months prior to leaving the country of origin?	Hours: minutes └─┴─┘ : └─┴─┘ hrs mins<br>Don't Know 91<br>Refused 92	**Q101**
	at present?	Hours: minutes └─┴─┘ : └─┴─┘ hrs mins<br>Don't Know 91<br>Refused 92	**Q102**
**EXPANDED: Physical Activit**			
**Sedentary behavior**			
The following question is about sitting or reclining at work, at home, getting to and from places, or with friends including time spent sitting at a desk, sitting with friends, traveling in car, bus, train, reading, playing cards or watching television, but do not include time spent sleeping.			
How much time do you usually spend sitting or reclining on a typical day<br><br>*(USE SHOWCARD)*	in the 12 months prior to leaving the country of origin?	Hours: minutes └─┴─┘ : └─┴─┘ hrs mins<br>Don't Know 91<br>Refused 92	**Q103**

Consider total time spent at work sitting, in an office, reading, watching television, using a computer, doing hand craft like knitting, resting etc. Do not include time spent sleeping.	at present?	Hours: minutes └─┴─┘ : └─┴─┘ hrs mins<br>Don't Know 91<br>Refused 92	**Q104**

CORE: History of Raised Blood Pressure			
**Question**		**Response**	
Have you ever had your blood pressure measured by a doctor or other health worker	in the 12 months prior to leaving the country of origin?	Yes 1<br>No 2 *If no, go to* ***Q116***<br>Don't Know 91 *If Don´t Know, go to* ***Q116***<br>Refused 92 *If refused, go to* ***Q116***	**Q105**
	at present?	Yes 1<br>No 2 *If no, go to* ***Q117***<br>Don't Know 91 *If Don´t Know, go to* ***Q117***<br>Refused 92 *If Refused, go to* ***Q117***	**Q106**
Have you ever been told by a doctor or other health worker that you have raised blood pressure or hypertension	in the 12 months prior to leaving the country of origin?	Yes 1<br>No 2 *If no, go to* ***Q116***<br>Don't Know 91 *If Don´t Know, go to* ***Q116***<br>Refused 92 *If Refused, go to* ***Q116***	**Q107**
	at present?	Yes 1<br>No 2 *If no, go to* ***Q117***<br>Don't Know 91 *If Don´t Know, go to* ***Q117***	**Q108**

		Refused 92 *If refused, go to* ***Q117***	
Were you first told		Yes 1<br>No 2<br>Don't Know 91<br>Refused 92	**Q109**
Have you taken any drugs (medication) for raised blood pressure prescribed by a doctor or other health worker?	in the 12 months prior to leaving the country of origin?	Yes 1<br>No 2<br>Don't Know 91<br>Refused 92	**Q110**
	at present?	Yes 1<br>No 2<br>Don't Know 91<br>Refused 92	**Q111**
Have you ever seen a traditional healer for raised blood pressure or hypertension	in the 12 months prior to leaving the country of origin?	Yes 1<br>No 2<br>Don't Know 91<br>Refused 92	**Q112**
	at present?	Yes 1<br>No 2<br>Don't Know 91<br>Refused 92	**Q113**
Have you taken any	in the 12 months	Yes 1	**Q114**

herbal or traditional remedy for your raised blood pressure?	prior to leaving the country of origin?	No 2<br>Don't Know 91<br>Refused 92	
	at present?	Yes 1<br>No 2<br>Don't Know 91<br>Refused 92	**Q115**

**CORE: History of Diabetes**			
**Question**		**Response**	
Have you ever had your blood sugar measured by a doctor or other health worker	in the 12 months prior to leaving the country of origin?	Yes 1<br>No 2 *If no, go to* ***Q129***<br>Don't Know 91 *If Don´t Know, go to* ***Q129***<br>Refused 92 *If refused, go to* ***Q129***	**Q116**
	at present?	Yes 1<br>No 2 *If no, go to* ***Q130***<br>Don't Know 91 *If Don´t Know, go to* ***Q130***<br>Refused 92 *If refused, go to* ***Q130***	**Q117**
Have you ever been told by a doctor or other health worker that you have raised blood sugar or diabetes	in the 12 months prior to leaving the country of origin?	Yes 1<br>No 2 *If no, go to* ***Q129***<br>Don't Know 91 *If Don´t Know, go to* ***Q129***<br>Refused 92 *If refused, go to* ***Q129***	**Q118**
	at present?	Yes 1<br>No 2 *If no, go to* ***Q130***	**Q119**

		Don’t Know 91 *If Don´t Know, go to* ***Q130***<br><br>Refused 92 *If refused, go to* ***Q130***	
Were you first told?		Yes 1<br><br>No 2<br><br>Don’t Know 91<br><br>Refused 92	**Q120**
Have you taken any drugs (medication) for diabetes prescribed by a doctor or other health worker	in the 12 months prior to leaving the country of origin?	Yes 1<br><br>No 2<br><br>Don’t Know 91<br><br>Refused 92	**Q121**
	at present?	Yes 1<br><br>No 2<br><br>Don’t Know 91<br><br>Refused 92	**Q122**
Have you taken insulin for diabetes prescribed by a doctor or other health worker	in the 12 months prior to leaving the country of origin?	Yes 1<br><br>No 2<br><br>Don’t Know 91<br><br>Refused 92	**Q123**
	at present?	Yes 1<br><br>No 2<br><br>Don’t Know 91	**Q124**

		Refused 92	
Have you ever seen a traditional healer for diabetes or raised blood sugar	in the 12 months prior to leaving the country of origin?	Yes 1<br>No 2<br>Refused 88	**Q125**
	at present?	Yes 1<br>No 2<br>Refused 88	**Q126**
Have you taken any herbal or traditional remedy for your diabetes	in the 12 months prior to leaving the country of origin?	Yes 1<br>No 2<br>Don't Know 91<br>Refused 92	**Q127**
	at present?	Yes 1<br>No 2<br>Don't Know 91<br>Refused 92	**Q128**

**CORE: History of Raised Total Cholesterol**			
**Question**		**Response**	
Have you ever had your cholesterol (fat levels in your blood) measured by a doctor or other health	in the 12 months prior to leaving the country of origin?	Yes 1<br>No 2 *If No, go to Q**140***<br>Don't Know 91 If *Don´t Know, go to Q**140***<br>Refused 92 *If Refused, go to* ***Q140***	**Q129**

<table>
<tr><td>worker</td><td>at present?</td><td>Yes 1
No 2 If No, go to Q141
Don’t Know 91 If Don´t Know, go to Q141
Refused 92 If refused, go to Q141</td><td>Q130</td></tr>
<tr><td rowspan="2">Have you ever been told by a doctor or other health worker that you have raised cholesterol</td><td>in the 12 months prior to leaving the country of origin?</td><td>Yes 1
No 2 If no, go to Q140
Don’t Know 91 If Don´t Know, go to Q140
Refused 92 If Refused, go to Q140</td><td>Q131</td></tr>
<tr><td>at present?</td><td>Yes 1
No 2 If no, go to Q141
Don’t Know 91 If Don´t Know, go to Q141
Refused 92 If Refused, go to Q141</td><td>Q132</td></tr>
<tr><td colspan="2">Were you first told?</td><td>Yes 1
No 2
Don’t Know 91
Refused 92</td><td>Q133</td></tr>
<tr><td rowspan="2">Have you taken any oral treatment (medication) for raised total cholesterol prescribed by a doctor or other health worker</td><td>in the 12 months prior to leaving the country of origin?</td><td>Yes 1
No 2
Don’t Know 91
Refused 92</td><td>Q134</td></tr>
<tr><td>at present?</td><td>Yes 1</td><td>Q135</td></tr>
</table>

		No 2<br>Don't Know 91<br>Refused 92	
Have you ever seen a traditional healer for raised cholesterol	in the 12 months prior to leaving the country of origin?	Yes 1<br>No 2<br>Don't Know 91<br>Refused 92	**Q136**
	at present?	Yes 1<br>No 2<br>Don't Know 91<br>Refused 92	**Q137**
Have you taken any herbal or traditional remedy for your raised cholesterol	in the 12 months prior to leaving the country of origin?	Yes 1<br>No 2<br>Don't Know 91<br>Refused 92	**Q138**
	at present?	Yes 1<br>No 2<br>Don't Know 91<br>Refused 92	**Q139**

CORE: History of Cardiovascular Diseases			
**Question**		**Response**	
Have you ever had a heart attack or chest pain from heart disease (angina) or a stroke (cerebrovascular accident or incident)	in the 12 months prior to leaving the country of origin?	Yes 1<br>No 2<br>Don't Know 91<br>Refused 92	**Q140**
	at present?	Yes 1<br>No 2<br>Don't Know 91<br>Refused 92	**Q141**
Have you taken aspirin regularly to prevent or treat heart disease	in the 12 months prior to leaving the country of origin?	Yes 1<br>No 2<br>Don't Know 91<br>Refused 92	**Q142**
	at present?	Yes 1<br>No 2<br>Don't Know 91<br>Refused 92	**Q143**
Have you taken statins (Lovastatin/Simvastatin/Atorvastatin or any other statin) regularly to prevent or treat heart disease	in the 12 months prior to leaving the country of origin?	Yes 1<br>No 2<br>Don't Know 91<br>Refused 92	**Q144**
	at present?	Yes 1<br>No 2<br>Don't Know 91	**Q145**

		Refused 92	

Mental Health/Suicide			
Have you seriously considered attempting suicide?	in the 12 months prior to leaving the country of origin?	Yes 1<br>No 2<br>Refused 92	**Q146**
	at present?	Yes 1<br>No 2<br>Refused 92	**Q147**
Did you seek professional help for these thoughts?	in the 12 months prior to leaving the country of origin?	Yes 1<br>No 2<br>Refused 92	**Q148**
	at present?	Yes 1<br>No 2<br>Refused 92	**Q149**
Have you made a plan about how you would attempt suicide	in the 12 months prior to leaving the country of origin?	Yes 1<br>No 2<br>Refused 92	**Q150**
	at present?	Yes 1<br>No 2<br>Refused 92	**Q151**
Have you ever attempted suicide?	in the 12 months prior to leaving the country of origin?	Yes 1<br>No 2<br>Refused 92	**Q152**

	at present?	Yes 1<br>No 2<br>Refused 92	**Q153**
What was the main method you used the last time you attempted suicide?<br><br>(SELECT ONLY ONE)	in the 12 months prior to leaving the country of origin?	Razor, knife or another sharp 1<br>Overdose of medication (e. g. 2<br>Poisoning with pesticides (e.g., rat 3<br>Other poisoning (e.g., plant/seed, household 4<br>Poisonous gases from charcoal 5<br>Other 6<br>Refused 92<br>Other (specify)	**Q154a**<br>**Q154**<br>**Q154c**<br>**Q154 d**<br>**Q154e**<br>**Q154f**<br>**Q154**<br>**Q154**
	at present?	Razor, knife or another sharp instrument 1<br>Overdose of medication (e. g. prescribed, over the-counter) 2<br>Poisoning with pesticides (e.g., rat poison, insecticide, weed-killer) 3<br>Other poisoning (e.g., plant/seed, household product) 4<br>Poisonous gases from charcoal 5<br>Other 6<br>Refused 92	**Q155a**<br>**Q155 b**<br>**Q155c**<br>**Q155 d**<br>**Q155e**<br>**Q155f**<br>**Q155**

		Other (specify)	g<br>Q155<br>h
Did you seek medical care for this attempt	in the 12 months prior to leaving the country of origin?	Yes 1<br>No 2<br>Refused 92	**Q156**
	at present?	Yes 1<br>No 2<br>Refused 92	**Q157**
Were you admitted to hospital overnight because of this attempt	in the 12 months prior to leaving the country of origin?	Yes 1<br>No 2<br>Refused 92	**Q158**
	at present?	Yes 1<br>No 2<br>Refused 92	**Q159**
Has anyone in your close family (mother, father,<br>brother, sister or children) ever attempted suicide?		Yes 1<br>No 2<br>Don't Know 91<br>Refused 92	**Q160**
Has anyone in your close family (mother, father,<br>brother, sister or children) ever died from suicide?		Yes 1<br>No 2<br>Don't Know 91<br>Refused 92	**Q161**

**CORE: Lifestyle Advice**			
**Question**		**Response**	
Have you visited a doctor or other health worker	in the 12 months prior to leaving the country of origin?	Yes 1<br>No 2 *If No and **Q1**=1, go to question **Q180** If No and **Q1**=2,*	**Q16<br>2**

		go to ***Q178***<br>Don't Know 91<br>Refused 92	
	at present?	Yes 1<br>No 2 *If no and* ***Q1****=1, go to question* ***Q180;*** *If No and* ***Q1****=2, go to* ***Q178***<br>Don't Know 91<br>Refused 92	**Q163**
During any of your visits to a doctor or other health worker **in the 12 months prior to leaving the country of origin**, were you advised to do any of the following? (RECORD FOR EACH)			
Quit using tobacco or don't start		Yes 1<br>No 2<br>Don't Know 91<br>Refused 92	**Q164**
Reduce salt in your diet		Yes 1<br>No 2<br>Don't Know 91<br>Refused 92	**Q165**
Eat at least five servings of fruit and/or vegetables each day		Yes 1<br>No 2<br>Don't Know 91<br>Refused 92	**Q166**
Reduce fat in your diet		Yes 1<br>No 2	**Q167**

	Don't Know 91<br>Refused 92	
Start or do more physical activity	Yes 1<br>No 2<br>Don't Know 91<br>Refused 92	**Q168**
Maintain a healthy body weight or lose weight	Yes 1<br>No 2<br>Don't Know 91<br>Refused 92	**Q169**
Reduce sugary beverages in your diet	Yes 1 *If **Q1**=1 go to **Q180***<br>No 2 *If **Q1**=1 go to **Q180***<br>Don't Know 91 If ***Q1**=1 go to **Q180***<br>Refused 92 *If **Q1**=1 go to **Q180***	**Q170**
During any of your visits to a doctor or other health worker **in the present**, are you advised to do any of the following? (RECORD FOR EACH)**)**		
Quit using tobacco or don't start	1<br>No 2<br>Don't Know 91<br>Refused 92	**Q171**
Reduce salt in your diet	Yes 1<br>No 2<br>Don't Know 91	**Q172**

	Refused 92	
Eat at least five servings of fruit and/or vegetables each day	Yes 1<br>No 2<br>Don't Know 91<br>Refused 92	**Q173**
Reduce fat in your diet	Yes 1<br>No 2<br>Don't Know 91<br>Refused 92	**Q174**
Start or do more physical activity	Yes 1<br>No 2<br>Don't Know 91<br>Refused 92	**Q175**
Maintain a healthy body weight or lose weight	Yes 1<br>No 2<br>Don't Know 91<br>Refused 92	**Q176**
Reduce sugary beverages in your diet	Yes 1 *If **Q1**=1 go to **Q180***<br>No 2 *If **Q1**=1 go to **Q180***<br>Don't Know 91 *If **Q1**=1 go to **Q180***<br>Refused 92 *If **Q1**=1 go to **Q180***	**Q177**

CORE (for women only): Cervical Cancer Screening			
The next question asks about cervical cancer prevention. Screening tests for cervical cancer prevention can be done in different ways, including Visual Inspection with Acetic Acid/vinegar (VIA), pap smear and Human Papillomavirus (HPV) test. VIA is an inspection of the surface of the uterine cervix after acetic acid (or vinegar) has been applied to it. For both pap smear and HPV test, a doctor or nurse uses a swab to wipe from inside your vagina, take a sample and send it to a laboratory. It is even possible that you were given the swab yourself and asked to swab the inside of your vagina. The laboratory checks for abnormal cell changes if a pap smear is done, and for the HP virus if an HPV test is done.			
Have you ever had a screening test for cervical cancer, using any of these methods described above?	in the 12 months prior to leaving the country of origin?	Yes 1<br>No 2<br>Don't Know 91<br>Refused 92	**Q178**
	at present?	Yes 1<br>No 2<br>Don't Know 91<br>Refused 92	**Q179**

Step 2 Physical Measurements

CORE: Blood Pressure		
**Question**	**Response**	
Interviewer ID	└─┴─┴─┘	
Device ID for blood pressure	└─┴─┘	
Cuff size used	Small 1<br>Medium 2<br>Large 3	
Reading 1	Systolic └─┴─┴─┘	**Q180a**

Record first measurement after the participant has<br>*rested for 15 minutes. Wait 3 minutes before taking*<br>*second measurement.*	(mmHg)	
	Diastolic (mmHg) └─┴─┴─┘	Q180b
Reading 2<br>*Record second measurement. Ask the participant*<br>*to rest for another 3 minutes before taking the*<br>*third measurement.*	Systolic (mmHg) └─┴─┴─┘	Q181a
	Diastolic (mmHg) └─┴─┴─┘	Q181b
Reading 3<br>*Record third measurement*	Systolic (mmHg) └─┴─┴─┘	Q182a
	Diastolic (mmHg) └─┴─┴─┘	Q182b
During the past two weeks, have been treated for raised blood pressure with drugs (medication) prescribed by a doctor or other health worker?	Yes 1<br>No 2<br>Don't Know 91<br>Refused 92	Q183
**CORE: Height and Weight**		
**For women:** Are you pregnant?<br>*If yes, skip to question*	Yes 1 If ***Yes, END***<br>No 2<br>Don't Know 91<br>Refused 92	Q184
Interviewer ID	└─┴─┴─┘	
Device IDs for height and weight	Height └─┴─┘<br>Weight └─┴─┘	
Height	in Centimetres (cm) └─┴─┴─┘.└─┘	Q185
Weight<br>*If too large for scale 666.6*	in Kilograms (kg) └─┴─┴─┘.└─┘	Q186
**CORE: Waist**		

Device ID for waist	└─┴─┘	
Waist circumference	in Centimetres (cm) └─┴─┴─┘ . └─┘	**Q187**
